# Assessment of Participation within the International Classification of Functioning, Disability, and Health (ICF): The Turkish Validity and Reliability of the Participation Scale

**DOI:** 10.1155/2021/6658773

**Published:** 2021-09-21

**Authors:** Onur Altuntaş, Esma Özkan, Barkın Köse, Orkun Tahir Aran, Meral Huri, Esra Akı

**Affiliations:** ^1^Department of Occupational Therapy, Faculty of Health Sciences, Hacettepe University, Ankara, Turkey; ^2^Department of Occupational Therapy, Gülhane Faculty of Health Sciences, University of Health Sciences Turkey, Ankara, Turkey

## Abstract

**Objective:**

The aim of the study was to investigate the reliability and validity of the Turkish version of the Participation Scale (P-Scale).

**Methods:**

A total of 152 students, with a mean age of 20.27 ± 2.19, participated in the study. Sociodemographic information (age, gender, and family income) was recorded; all participants completed the P-Scale twice with a 15-day interval. Translation and cross-cultural adaptation were performed to analyze the validity and reliability of the P-Scale. Cronbach's alpha and McDonald's alpha were used for scale reliability statistics and explanatory (EFA) and confirmatory (CFA) factor analysis; Mardia's multivariate normality and assumption tests were used for the validity of the scale. The factor extraction methods were minimum rank factor analysis in EFA and weighted least squares mean and variance adjusted estimator polychoric correlation matrix in CFA.

**Results:**

Internal consistency of the scale was found good with Cronbach's alpha (0.852) and excellent with McDonald's alpha (0.924). The EFA and CFA resulted in two-factored structure, with the explained variance found to be higher than 30%.

**Conclusions:**

Analysis demonstrated that the P-Scale had a satisfactory level of reliability and validity in Turkish university students.

## 1. Introduction

Participation is defined by the World Health Organization as “involvement in a life situation” [1]. A daily life situation means a person's interaction and participation in areas of normal living or community life. This includes social, economic, civic, interpersonal, domestic, and educational domains of daily living, most of which concern every person. Participating in daily life activities is a vital part of human development and life experience. An individual acquires skills and competencies, establishes communication with others and society, and develops meaning and goals in his/her life [2].

Participating in work, school, and social life has a positive impact on health and wellbeing [2]. It also has an important role for adults in terms of life satisfaction, physical and mental health, and the development of social networks [3]. Besides the importance of participation in terms of physical and psychological health, in cases of lack of participation, bad health conditions and lack of wellbeing are seen to result. In addition, occupational disruption and deprivation give rise to participation problems, in particular among the unemployed, individuals of lower socioeconomic status, refugees, minorities, and disabled individuals [2]. Moreover, transactions in life circles, such as attending university, adulthood, and marriage, may change individuals' participation.

University life constitutes a period in which the vital change of leaving adolescence and entering adulthood occurs. In university life, individuals quit the environments that their family provided and determined for them and are confronted with different opinions and surroundings. Reduced family supervision, the emergence of a new social circle, an increase in academic and financial responsibilities, gaining self-discipline, career choice, and having autonomous functioning are common experiences in this period [4–6]. Differences in socioeconomic classes in university life may cause some students to fall behind in terms of academic and social integration [7]; conversely, social integration aids academic performance and maintenance [8]. This situation provides better learning, cognitive development, critical thinking, and personal and moral development. Participation and extracurricular activities reduce the proportion of behavioral and emotional breakdowns and dropouts in school-aged children with psychological problems and strengthen their relationships in school and between friends [9–12].

There are several instruments developed to assess participation restrictions, such as the Activity and Participation Questionnaire [13], the Participation Assessment with Recombined Tools-Objective [14], the Participation Profile [15], the International Classification of Functioning (ICF), and the Disability and Health Measure of Activity and Participation-Screener (IMPACT-S) [16]. The Participation Scale (P-Scale) is one of the more recent participation measurements and is based on ICF participation domains. The ICF divides participation into subdomains: learning and applying knowledge, domestic life, communication, mobility, self-care, interpersonal interactions, major life areas like school and work, community, civilian, and social life [1].

Noonan et al. stated that instruments that intend to measure participation cover six to eight domains of the ICF [17], and the P-Scale covers eight out of nine domains [18]. Additionally, the P-Scale is one of the recent participation measurements that assess the impact of stigma on social participation [19].

The P-Scale measures individuals' perceived constraints. Even though people have similar health conditions, they may experience very different levels of participation constraints; therefore, individual perception is very important [20, 21]. As participation constraint is experienced by people in various conditions of health, it is very important that it does not include items pertaining to illness. With its “comparison to peers” concept, the P-Scale enables an individual to compare himself/herself with a peer who is in a similar situation in terms of sociocultural, economic, and demographic aspects besides illness and disability [20, 22]. The Participation Scale has been translated into many languages, including Nepalese, Indian, Brazilian, Amharic, Arabic, and Chinese [23–26].

The P-Scale is a tool for assessing problems perceived in main socioeconomic living spaces and is based on 18 items [20]. The questions in the scale measure specific aspects of ICF activity and participation domains, including learning and applying knowledge, general tasks and demands, communication, mobility, self-care, domestic life, interpersonal interactions and relationships, major life areas and community, and social and civic life. The scale uses a 5-point grading system (0—no restriction; 1—some restriction, but no problem; 2—small problem; 3—medium problem; and 5—large problem). By summing the item scores, a total score range of 0–90 is obtained. This final score can be converted to participation constraint scores. Possible grades are no important constraint (0–12), mild restriction (13–22), moderate restriction (23–32), severe restriction (33–52), and extreme restriction (53–90). The possible grades of the scale were determined by Van Brakel et al. according to distribution of the scores in the control group and client populations [18, 20]. Stevelink et al. [27, 28] investigated the factor structure of the P-Scale in two studies. They stated that the P-Scale has a two-factored structure and showed good fit to the factors; there was also a good correlation between the factors. The two factors were named “work-related participation” and “general participation.”

Young people in Turkey sit a challenging exam to get the opportunity to enter university. During university life, there are expectations such as ensuring social, academic, and intellectual development, finding adequate scholarships and housing opportunities, successfully graduating, and finding employment after graduation. However, university life is the most important transitional phase of students' lives, where they encounter difficult emotions such as anxiety, stress, and sadness and try to find strategies to deal with them [29]. The necessity of being in different cultural environments, changing conditions, adaptation to university, economic difficulties, accommodation, and environmental variables can negatively affect the lives of students during this process [29]. University life is not only about educating oneself but also supporting students in terms of social life, employment, self-efficacy, social support, independence, and wellbeing. All these aspects are related to ICF participation domains.

In a study by the Centre for the Study of Higher Education investigating the reasons for low-income youth's continued low participation in higher education, they reported that low educational attainment in school, low educational aspirations, and low school completion rates affect outcomes in continuing education. In addition, this study concluded that such people are less optimistic about getting into a university, have less confidence in their personal and career relationships with higher education, and are more likely to be alienated from the cultures of universities [30, 31]. According to the study conducted by Keskin et al., the socioeconomic status of the families affects the university education and training success of the students and their academic/professional choices [32].

In a study by Polesel, it was stated that financial reasons have a significant effect on the decision to postpone in university life. The researcher emphasizes that the costs of travel and living away from home are factors in young people's decision to postpone [33]. The study by Wilks and Wilson found that financial disadvantage has a significant impact on the overall educational experience of students from low socioeconomic backgrounds, influencing students' aspirations, choices, and overall decisions about their participation in higher education [34].

Students of low socioeconomic status often deal with financial problems that significantly affect their success in higher education [35]. These financial problems can negatively affect a person's life in many areas, from academic success to continuing education at university. A comprehensive assessment tool is needed in order to accurately identify the difficulties experienced by students whose lives are negatively affected by low income and who have problems in participation and to produce constructive solutions. We aimed to carry out a validity and reliability study of the P-Scale according to Turkish culture, which we think will be useful in revealing the participation of students with low monthly income in university life according to ICF domain areas. Therefore, we conducted a validation and reliability study of the Turkish P-Scale that evaluates the ICF participation domains.

## 2. Methods

### 2.1. Participants

Brochures explaining the study were hung in the different areas of the university, such as cafeterias and library. Volunteering students who fit the inclusion criteria were then selected for the study. In this study, since we want to reveal the suitability of an assessment tool to Turkish culture in order to reveal whether the low income of healthy people with no disabilities will affect their participation, the most important inclusion criterion is having a subminimum monthly wage family income (1,603.12 TL; 233 US Dollars; 208.83 Euros). The other inclusion criteria were being a university student and volunteering to participate in the study. However, students with chronic disabilities or chronic neurological or orthopedic disorders were excluded from the study.

Gorsuch suggests that sample size for explanatory factor analysis would be 5–10 participants for each item and should not be lower than 100 participants in total; Kline suggests that the sample size should be 100–200 for confirmatory factor analysis. In this study, a total of 184 individuals fulfilled the inclusion criteria, 173 of whom agreed to participate. Partially completed questionnaires were excluded. Although attempts were made to obtain complete data from all those participants, only 152 participants provided complete data that could be included in the statistical analysis. Sociodemographic information (age, gender, and monthly family income) was taken from all participants.

Before their participation, written and oral informed consent was obtained from all subjects. This study was approved by the University Noninterventional Clinical Researches Ethics Board and was conducted in accordance with the rules of the Declaration of Helsinki. The questionnaires were given at the university in 2019, between January and May. The students were tested within twice over two weeks to assess test–retest reliability.

### 2.2. Translation Procedure

The translation process of the original English version of the P-Scale was conducted according to the guidelines of Guillemin et al. [36] and guidelines described in the P-Scale manual [18]. Two researchers who had at least 18 years' experience in occupational therapy (and were native Turkish speakers) separately translated the original English version into Turkish. The two Turkish translations were compared for inconsistencies, finalizing the first step of the translation procedure. The output was translated back to English by an English-native professional translator, blindly and independently. This translator neither had any medical knowledge nor knew anything about the P-Scale. The back-translated version was compared with the original English version and sent to the scale creator (Van Brakel) for approval and received an approval for no inconsistencies. Additionally, the latest Turkish version was sent to 40 students for debriefing of the scale. A three-point Likert scale was applied to the question of whether the Turkish version was understandable or not; all 40 participants answered “totally understandable.” The translations were similar, and the final Turkish P-Scale was matched to the English P-Scale questionnaire. An expert panel meeting of all authors was held to consider the final version of the P-Scale. The authors discussed and considered potential changes to the translated introduction and items.

### 2.3. Statistical Analysis

The P-Scale and sociodemographic data was checked for mistakes and transferred to SPSS for Windows, version 23.00. For descriptive data, numbers and percentages are given as descriptors and indicate standard deviation for normal distributed data.

Psychometric properties of the P-Scale were assessed with reliability and validity analyses. Before conducting these analyses, item contribution to the scale structure was analyzed by item-total scale correlation and Cronbach alpha analysis. Pearson correlation coefficient was used for item analysis. The scale's reliability was analyzed with Cronbach's alpha score (*α*) and McDonald's alpha score (*ω*); interclass correlation coefficient (ICC) and weighted kappa were used for test–retest reliability. Exploratory (EFA) and confirmatory (CFA) factor analyses were used to analyze construct validity. Kaiser–Meyer–Olkin (KMO) and Bartlett sphericity tests were used to assess data suitability for factor analysis. Mardia's multivariate normality test and Mardia's multivariate normality assumption tests were checked to determine factor extraction method in EFA and parameter assumption method in CFA [37]. Since these assumptions were not met, minimum rank factor analysis (MRFA) in EFA and weighted least squares mean and variance adjusted estimator (WLSMV) polychoric correlation matrix were used together [38, 39]. MRFA was used as a factor extraction method in EFA [38]. A polychoric correlation matrix was used to collect correct, efficient, and reliable parameter assumptions [40]. Promin rotation, one of the oblique rotation methods, was used to reveal an understandable and easy-to-interpret factor structure [41]. The following factor number decision methods were used: Kaiser criterion [42], explained variance ratio, Velicer's minimum average partial (MAP) test combined with the parallel analysis [43], and least-averaged partial correlation [40, 44]. First-order CFA was used. Model fit indexes were used to determine the best suitable analysis model for the dataset and theoretical model. Robust parameter estimation methods and polychoric correlation were used in order to avoid the effect of the floor effect, which disrupts normal distribution on the exploratory and confirmatory factor analysis. SPSS for Windows version 21.00 was used to analyze data, FACTOR 10.8.04 was used for EFA, and Mplus package 6.12 was used for CFA. Statistical significance level was set on alpha 0.05.

## 3. Results

### 3.1. Descriptive Statistics

Of the 152 students who completed the test and retest assessments, the mean age was 20.27 ± 2.19 years (min =18, max =32). 132 (86.8%) of the students were female, and 20 (13.2%) were male. 132 (86.8%) of the participants were occupational therapy students, and 20 (13.2%) of the participants were physical therapy students. The majority of the students were living in a dorm (58.6%, *n* = 89) or living with parents (21.7%, *n* = 33) (Table 1).

### 3.2. Reliability of the P-Scale

The scale's reliability was found to be good with regard to the 0.852 Cronbach alpha score, which was higher than 0.70, as suggested by Nunnally [45].  Table 2 shows that the 14^th^ (0.219) and 16^th^ (0.250) items' item-total correlation scores were lower than 0.300, as Nunnally suggested [45]. However, Cronbach's alpha score did not change after removing these items (0.853 and 0.852, respectively). Therefore, it was decided whether the items would remain or be removed after their contribution to the scale and CFA.

When the items' distribution was investigated, no items had a ceiling effect (<15%), but all the items had a floor effect (>15%).

### 3.3. Exploratory Factor Analysis

EFA was used to analyze the structural validity of the P-Scale. The KMO test score was 0.788, the Bartlett test of sphericity score was 870.40, and the determinant value was 0; 00238 > 0.00001, indicating that the data was suitable for factor analyses [46].

Mardia's test of multivariate normality was used to analyze the normal distribution of the P-Scale's 18 items. The data's skewness and kurtosis values were 0.764–3.891 and 0.644–16.380, respectively. According to Mardia's test, the data was not normally distributed because of significant (*p* < 0.001) multivariate kurtosis coefficients (586.773).

**Figure 1:**
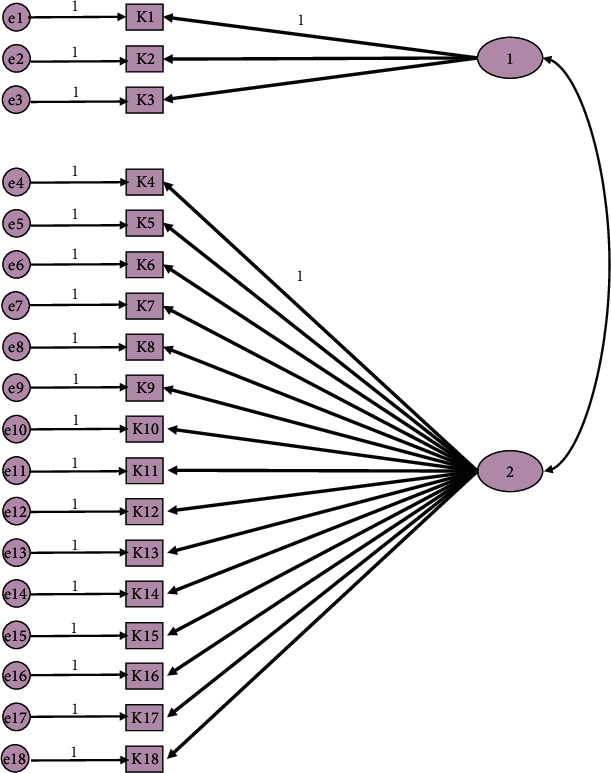
Parallel analysis models.

Table 3 shows factor structure analysis reports, in which factor loads are in the range of 0.327–0.951 for the first factor and between 0.330 and 0.992 for the second factor. Factor loads were higher than 0.32, as suggested by Tabachnick et al. [47]. The eigenvalues of the two-factor structure obtained by MRFA factor subtraction method were 5.91 and 1.57, and the variance explanation rate was 45.85%. MAP results were found to be one factor, and parallel analysis revealed a factor of two with a 95% confidence level (Figure 1). This factor model did not have a simple structure as suggested by Thurstone [48], and it was evaluated by Bentler simplicity statistics (0.937) [49]. Factor loads are in the range of 0.330–0.992 for the second factor. Factor loads were higher than 0.32, as suggested by Tabachnick et al. [47].

In order to measure the stability of the repetitions, the test was readministered after 15 days, and the scale was applied to 152 people. Weighted kappa values for the test were 0.625 ± 0.065 for factor 1, 0.791 ± 0.041 for factor 2, and 0.804 ± 0.039 for scale total. Intraclass correlation coefficient (ICC) values were 0.639 for factor 1, 0.816 for factor 2, and 0.834 for total. Weighted kappa values were found to be 0.756 ± 0.049 for factor 1 and 0.732 ± 0.052 for factor 2 (found via MRFA). ICC values were 0.770 for factor 1 and 0.745 for factor 2.

### 3.4. Confirmatory Factor Analysis

Since the data obtained from the Likert-type P-Scales are ordinal and far from multivariate normality, a mean and variance correction parameter estimation method (weighted least squares mean and variance adjusted estimator (WLSMV)) developed by Muthén [39] was used as the parameter estimation method in CFA. Primary level CFA was applied with a polychoric correlation matrix to obtain accurate, consistent, effective parameter estimates [50].

The P-Scale's (*χ*^2^)/df ratio shows good agreement with 1.622. Goodness-of-fit indices of TLI (0.932), RMSEA (0.064), and WRMR (0.927) were within acceptable limits, while CFI (0.940) showed moderate compliance (Table 4).

**Figure 2:**
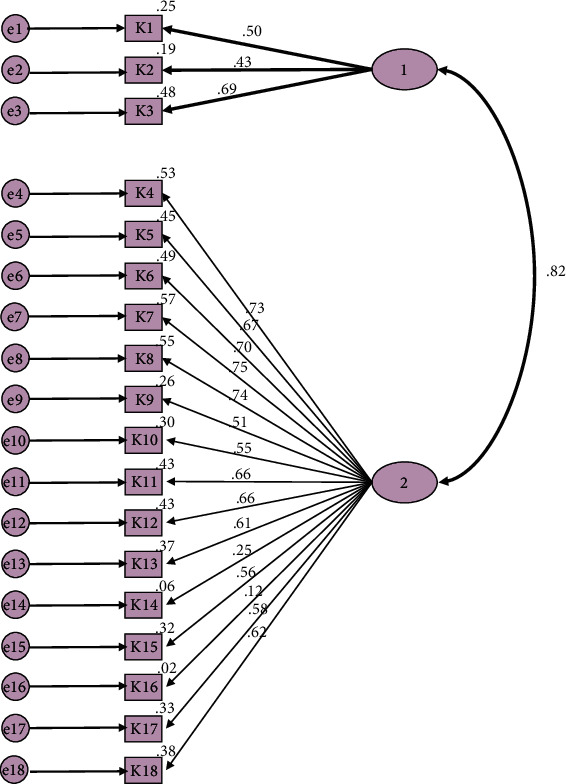
Confirmatory factor analysis model.

When standardized factor loads obtained by the WLSMV parameter estimation method of two-factor uncorrected primary level measurement model were examined, they were found to be in the range of 0.616–0.690 (Item 1–Item 3) for factor 1 and 0.40–0.860 (Item 4–Item 18) for factor 2. The *t* values of all items were found to be greater than 1.96 and significant at 0.05 significance level. The Cronbach alpha internal reliability coefficients for the factors were 0.591 and 0.837, respectively. Composite reliability (McDonald's (*ω*) alpha coefficient) was found to be 0.690 and 0.927, respectively (Table 5, Figure 2).

## 4. Discussion

This study is aimed at investigating the validity and reliability of the P-Scale. We found that the Turkish P-Scale was a valid and reliable scale. In this cross-cultural adaptation of the P-Scale, there were no changes made to the scale, and the expert panel version was accepted as it was. Also, the debriefing answers showed that the P-Scale was understandable for the examinees. We believe that the P-Scale was successfully adapted to the Turkish language and that this scale is suitable for use in the Turkish university context.

### 4.1. Reliability

The internal consistency measured with Cronbach's alpha was 0.93 for the original P-Scale [20, 23], the Indonesian version's Cronbach's alpha was 0.70–0.83 [51], and the Chinese version's Cronbach's alpha was 0.93 [26]. The Igbo version's Cronbach's alpha was 0.91 [52]. The Turkish P-Scale had a Cronbach's alpha score of 0.852. Cronbach's alpha score is only consistent when there is equality in the scale items' means and variance, accepted multivariate normality assumption, and univariate scale structures. According to Guttman, Cronbach's alpha score shows the base value of reliability rather than reliability itself. This score is not the only score that is used for reliability, and it is insufficient for reliability analysis in many circumstances [53]. Therefore, McDonald's alpha score, which analyzes reliability coefficients from a factor analysis perspective, was used and was found to be 0.92. We believe the difference in alphas was caused by the sample group differences. All previous studies had a sample group consisting of people with various disabilities; our study consisted of participants who were university students with low income.

In this study, the intertester reliability coefficient was 0.90. Finding a high reliability coefficient of the P-Scale demonstrates that reliability was at a good level [27]. When the fact that the anticipated reliability level is 0.70 for the assessment tools is considered, the findings obtained towards determining the reliability of the P-Scale proved that the scale is reliable at a sufficient level. Thammaiah et al. reported that they meaningfully repeated tests two weeks later [54]. Similarly, in the present study, the Turkish version test–retest scores (ICC) were meaningful two weeks later.

### 4.2. Construct Validity

Factor analysis provides a diagnostic tool to evaluate the collected data that is in line with the theoretically expected pattern or structure of the target construct and thereby determines if the measures used have indeed measured what they are purported to [49]. In the study conducted by Stevelink et al., three different factor structures (one-, two-, and four-factor structure) were reported [27]. They stated that the P-Scale has a two-factored structure and showed good fit to the factors; there was also a good correlation between the factors. The two factors were named “work-related participation” and “general participation.” In our study, we found a two-factored structure similar to Stevelink et al.'s study.

Many students try to make their way into university life each year by passing a rigorous exam and scoring above a certain threshold. In this race, the children of low-income families lack the guidance and support they need to prepare for university, apply for the most suitable schools, apply for financial aid, enroll and continue their studies, and, finally, graduate. As a result, there are great differences in educational achievement between students from low-income families and their high-income peers [29, 55]. We believe it is important to remove inequality between students in university life. The P-Scale is a valid and reliable scale, and we believe it is possible to determine students with participation limitations and provide guidance for scholarship applications and plan summer schools and guidance through their university life.

### 4.3. Limitations of the Study

This study has several limitations. (1) Our sample group only consisted of university students with low income; other studies included participants with disabilities. We believe this is a minor limitation, because low income also affects participation and social life. Additionally, Van Brakel [18] stated that the P-Scale is also able to assess participation without concerning health issues. We believe that this specification of the scale removes this limitation. (2) There are no assessments that are valid and reliable and assess participation in Turkish language. Therefore, we could not analyze external (convergent) validity. (3) This study is based on the classic theory test, and we suggest future research using item response theory, which can provide more detailed information about the scale's item difficulty, meaningful scaling of latent variables, discriminative ability, and so on. (4) We think that the P-Scale is a state-like scale [56] that generally evaluates the experiences perceived by the examinee. However, we suggest a revision to the P-Scale to include trait items that evaluate the characteristics of the examinee, since we believe personal characteristics might affect the participation of the individual. (4) The numbers of male and female participants in this study are not equal. However, gender distribution in Turkish universities is nearly equal between the genders. We think that female participants tend to share their problems more than males, and because of this, we have higher numbers of female participants. However, we suggest further studies to examine the difference between males and females in the Turkish context, which is not one of our study hypotheses. (5) We used the same participants' data for both CFA and EFA; due to the number of participants in the study, we were not able to divide the dataset, but we applied both tests to all participants.

## 5. Conclusion

All the results obtained from the validity and reliability study of the Turkish P-Scale proved that the scale has validity and reliability at a sufficient level to evaluate the participation of university students with low incomes. By using this scale, it will be ensured that the participation levels of low-income students at university will be monitored and strategies to increase their participation will be provided. By ensuring the participation of these students in university life, successful, enthusiastic, and strong generations will be raised.

## Figures and Tables

**Table 1:** Sociodemographic properties of the participants.

Gender	Female	132 (%86.8)
	Male	20 (%13.2)
Age (years) (mean ± standard deviation) (min–max)		20.27 ± 2.19 (18–32)
Division	Physical therapy	20 (%13.2)
	Occupational therapy	132 (%86.8)
Accommodation	Family	33 (%21.7)
	Alone	6 (%3.9)
	Friends	24 (%15.8)
	Dorm	89 (%58.6)

**Table 2:** Item-total correlation.

	Items	Mean when item deleted	Total variance when item deleted	Item-total correlation	Squared multiple correlation	Cronbach's alpha when item deleted
*N* = 152 Cronbach alpha = 0.852	1	12.3684	124.711	0.441	0.339	0.847
	2	12.4211	128.894	0.398	0.370	0.848
	3	12.1776	127.922	0.471	0.352	0.844
	4	12.7961	130.137	0.510	0.590	0.842
	5	12.9276	130.306	0.534	0.608	0.842
	6	12.7697	128.867	0.513	0.464	0.842
	7	12.6908	128.189	0.540	0.531	0.841
	8	13.1053	129.962	0.542	0.445	0.841
	9	13.0132	130.079	0.465	0.383	0.844
	10	12.8355	127.158	0.487	0.372	0.843
	11	12.6316	129.188	0.535	0.465	0.841
	12	13.2500	133.315	0.545	0.576	0.843
	13	12.8750	133.567	0.433	0.444	0.846
	14	13.0197	138.867	0.219	0.158	0.853
	15	12.9737	131.205	0.425	0.332	0.846
	16	13.2303	138.576	0.250	0.294	0.852
	17	12.0592	124.003	0.465	0.370	0.845
	18	12.5789	123.848	0.512	0.379	0.842

**Table 3:** Factor loads of two-factor structure obtained by MRFA and factor subtraction method.

	Factor 1	Factor 2	Skewness	Kurtosis
Item 1	0.172	0.315	1.193	0.021
Item 2	0.306	0.057	1.293	0.832
Item 3	0.630	-0.180	1.014	0.458
Item 4	0.715	-0.001	1.788	3.087
Item 5	0.756	0.051	2.332	6.118
Item 6	0.445	0.217	1.553	1.686
Item 7	0.379	0.328	1.262	0.628
Item 8	0.153	0.662	2.911	8.143
Item 9	-0.035	0.754	2.575	5.935
Item 10	0.092	0.584	2.083	3.313
Item 11	0.680	-0.033	1.175	0.823
Item 12	0.464	0.452	3.804	15.721
Item 13	0.951	-0.378	1.283	0.272
Item 14	-0.079	0.378	2.080	4.498
Item 15	-0.049	0.690	2.326	4.768
Item 16	-0.504	0.992	3.904	16.840
Item 17	0.333	0.139	0.767	-0.641
Item 18	0.235	0.434	1.555	1.188
Eigenvalue	5.91	1.57		
Skewness	0.764	3.891		
Kurtosis	0.644	16.380		
DVR^∗^	36.25%	9.60%		
Total DVR^∗^	45.85%			
MAP^∗∗^	1 factor			
PA^∗∗∗^	Parallel analysıs (PA) based on principal component analysis Advised number of dimensions when 95 percentiles are considered: 2 Advised number of dimensions when mean is considered: 4			
Cronbach's alpha	0.801	0.704		
ICC^∗∗∗∗^	0.770	0.745		
Weighted kappa	0.756 ± 0.049	0.732 ± 0.052		

*n* = 152. ^∗^Described variance ratio. ^∗∗^Minimum average partial. ^∗∗∗^Parallel analysis. ^∗∗∗∗^Intraclass correlation coefficient.

**Table 4:** Goodness-of-fit indexes.

Model fit	Good fit	Acceptable fit	Average fit	Results for P-Scale *N* = 152
*χ*^2^/sd (*p*)	0 ≤ *χ*^2^ ≤ 2	2 < *χ*^2^ ≤ 5		217.343/134 = 1.622 *p* < 0.001
RMSEA 95% CI	0 ≤ RMSEA ≤ 0.05	0.05 < RMSEA ≤ 0.08	0.08 < RMSEA ≤ 1.00	0.064
CFI	0.97 ≤ CFI ≤ 1.00	0.95 ≤ CFI < 0.97	CFI > 0.90	0.940
TLI	0.95 < TLI ≤ 1.00	0.90 ≤ TLI ≤ 0.95		0.932
WRMR		WRMR < 1.00		0.927

*χ*^2^/sd: chi square; RMSEA: the root mean square error of approximation; CFI: confirmatory fit index; TLI: Tucker-Lewis index; WRMR: weighted root mean square residual.

**Table 5:** Standard factor loads for the original two-factor unadjusted model.

Item	Factor 1 loadings	Factor 2 loadings	SD	*t*	*p*
Item 1	0.690		0.069	9.996	<0.001
Item 2	0.616		0.075	8.186	<0.001
Item 3	0.659		0.075	8.848	<0.001
*Cronbach alpha coefficient forfactor* 1 = 0.591						
*Composite reliabilitycoefficient* = 0.690						
Item 4		0.745	0.044	16.877	<0.001
Item 5		0.826	0.036	22.819	<0.001
Item 6		0.684	0.053	12.824	<0.001
Item 7		0.715	0.045	15.875	<0.001
Item 8		0.770	0.064	11.935	<0.001
Item 9		0.682	0.070	9.777	<0.001
Item 10		0.656	0.063	10.386	<0.001
Item 11		0.689	0.048	14.285	<0.001
Item 12		0.860	0.056	15.453	<0.001
Item 13		0.644	0.054	11.972	<0.001
Item 14		0.401	0.079	5.086	<0.001
Item 15		0.649	0.061	10.585	<0.001
Item 16		0.526	0.074	7.077	<0.001
Item 17		0.579	0.061	9.519	<0.001
Item 18		0.678	0.057	12.000	<0.001
*Cronbach alpha coefficient forfactor* 2 = 0.837						
*Composite reliabilitycoefficient* = 0.927						

SD: standard deviation.

## Data Availability

The data are not publicly available. Please contact the corresponding author for information on access to data.
